# The effect of structured stepwise presentations on students’ fraction learning: an eye-tracking study

**DOI:** 10.3389/fpsyg.2023.1125589

**Published:** 2023-05-12

**Authors:** Xiaoqing Shang, Rangmei Li, Yangping Li

**Affiliations:** ^1^Faculty of Education, Shaanxi Normal University, Xi’an, Shaanxi, China; ^2^School of Mathematical Sciences, Beijing Normal University, Beijing, China; ^3^Hangzhou International Urbanology Research Center and Zhejiang Urban Governance Studies Center, Hangzhou, Zhejiang, China

**Keywords:** structured presentation, stepwise presentation, attention guidance, eye-tracking, fraction learning

## Abstract

The structured stepwise presentation is based on the segmenting and cueing principles. The main purpose of the study was to examine the effect of the structured stepwise presentations on students’ attention and fraction learning. A total of 100 primary pupils participated in this study. They were divided into three parallel groups and were, respectively, applied three kinds of presentation types (structured and stepwise, no structure and stepwise, and structure and no stepwise) of the teaching content to learn the fraction concept. A stable eye tracker was used to record students’ visual attention during learning, the first fixation duration and total fixation duration of students were recorded, and the regression time was also calculated within correspondent relative elements. After the experiment, through a one-way ANOVA test, we found significant differences among the three groups in students’ attention. The learning performance of these three groups also differed. The results showed that structured stepwise presentation played an important role in attention guidance during fraction teaching. It better guided students’ attention to connecting relative elements and resulted in better learning performance in fraction learning. The findings suggested the importance of structured stepwise presentations during teaching practices.

## 1. Introduction

Multimedia technology provides opportunities to optimize teaching and learning due to its multiple and dynamic information presentation features. Especially for primary school teaching, presentations based on multimedia technology are beneficial for promoting deep understanding, since abstract concepts can be illustrated in multiple methods and visualized by demonstrating dynamic sequential processes (Rieber, 1990; Park and Hopkins, 1992), which draw students’ attention to key information, thus effectively prevent them from being distracted by external factors (Boucheix and Guignard, 2005; Wouters et al., 2008).

Cueing and segmenting in multimedia presentations have been successfully used to guide students’ attention and promote their learning (De Koning et al., 2007; Mayer, 2014; Van Gog, 2014; Rey et al., 2019). Cueing refers to the manipulation of visuospatial characteristics of instructional materials (Mautone and Mayer, 2001), such as distinctive colors, arrows, labels, picture references, and mode of arrangement, which can draw students’ attention to related content and decrease the associated extraneous cognitive load (De Koning et al., 2009; Wang et al., 2013). Segmenting refers to the process of breaking the instructional content into individual parts (Rey et al., 2019). Combining cueing with segmenting to reflect structural relationships and framework of knowledge is called structured presentations in our study (De Koning et al., 2009), which can enable students to connect corresponding information easily. In addition, stepwise is a kind of dynamic cue based on segmentation, which is used to gradually highlight each segment accompanied by oral guidance (Chen et al., 2016). The stepwise presentation allows students to learn at their own pace and gives them sufficient time to integrate information, thus reducing and compensating for potential split attention (Stiller et al., 2011; Rey et al., 2019). Therefore, guiding students’ attention through multimedia techniques is key to optimizing students’ learning.

Research has consistently shown that fractions are challenging for primary students to learn (Lin et al., 1996; Lortie-Forgues et al., 2015; Reinhold et al., 2020). It is hard for students to make connections between natural numbers and fractions, as the fraction concept involves high-element interactivity (Reinhold et al., 2020). The inappropriate teaching designs, such as scenario fiction, formalization of process, and improper connection of prior knowledge, further increase students’ cognitive load and make them feel struggling to understand (Lin et al., 1996). To our knowledge, research on combining structured presentations and stepwise presentations to guide students’ attention and its impact on students’ fraction learning, remains unexplored. Eye-tracking technology can scientifically interpret learners’ learning processes through their eye movements, and, especially in multimedia learning, fixation duration and regression time are key indicators of students’ attention and cognitive processes (Ozcelik et al., 2010; Jamet, 2014; Jian et al., 2019; Pi et al., 2020). Therefore, in this study, we attempted to employ eye-tracking technology to investigate the effect of teaching presentations on students’ learning of fractions by integrating structured presentations and stepwise presentations into teaching to attract students’ attention.

## 2. Theoretical background

### 2.1. Cues and segmentation adjust students’ cognitive load

People’s psychological resources are limited, and they cannot deal with excessive amounts of information simultaneously. The greater the visual difference between a perceived object and the background, the more noticeable and more easily it is perceived by an individual (Treisman and Schmidt, 1982). For fraction learning with high-element interactivity, simply using text sequence descriptions is difficult to provide the visual difference for students, and would waste students’ cognitive resources to visualize information by themselves (Seufert and Brünken, 2006; Richter et al., 2016). According to the cueing principle, cues can decrease students’ extraneous cognitive load and draw their attention to key teaching points (Seufert and Brünken, 2006; Doolittle and Altstaedter, 2009; Van Gog, 2014). The empirical studies showed that the total and first fixation durations of students on signal presentations were longer than on the no-signal teaching materials (Boucheix and Lowe, 2010; Ozcelik et al., 2010; Pi et al., 2020). Thus, adding cues to the text presentations of fraction concepts could strengthen students’ retention of knowledge and promote learning efficiency (Wang et al., 2013; Schneider et al., 2018).

Segmenting–a form of temporal cueing–involves hidden signals that increase the salience of the natural boundaries between events in a process. Unblocked information presentation confuses key and background information, hinders students from selecting and organizing key information, and results in cognitive overload, reducing students’ available cognitive capacity for dealing with essential information (Cierniak et al., 2009; Mayer and Pilegard, 2014). The principle of segmenting holds that complex content should be broken into smaller, manageable, learner-controlled units (Mayer and Pilegard, 2014) to maximize students’ limited working memory. Segmenting in a presentation can reduce the viewers’ cognitive processing burden (Kurby and Zacks, 2008; Spanjers et al., 2010) and decrease their extraneous cognitive load, because the information is clearly organized and minimizes the time spent searching for related information. Therefore, teaching presentations organized by segmentation enable learners to extract key information effectively, deeply understand the content at their own pace (Rey et al., 2019), and avoid split attention.

Segmentation can also balance students’ intrinsic cognitive load by organizing multiple types of information (Spanjers et al., 2010; Rey et al., 2019), especially when presenting intricate content. The concept of fractions comes from daily life and undergoes a transformation from life context to mathematical symbols, so the rich information presented in different ways could arose students’ learning interests and facilitate their understanding (Mitchell and Miller, 2010). However, teachers often transmitted the fraction concept directly to students, instead of connecting real-life scenarios with it (Lin et al., 1996). Using segmentation, the fraction concept can be broken into simple components, with sub-blocks to represent sub-concepts related to key content that students can learn and recall more easily, enabling them to experience organizing individual segments. At the same time, multiple representations including symbols and words can be used to express the fraction concept (Obersteiner et al., 2015; Reinhold et al., 2020). Thus, segmentation is beneficial for reserving cognitive capacity to enhance the perception of essential information, providing a method for learning fractions.

### 2.2. Structured presentations provide cues to draw attention to segments

In terms of multimedia techniques, structured presentations usually combine cueing and segmenting. First, segmenting splits complex content into known content, clearly distinguishing each sub-concept for identification by students. Cueing further reveals the logic of knowledge generation by building bridges between blocks and organizing each block into a coherent representation (De Koning et al., 2009). Combining cueing with segmenting supports the selection and organization of learning content, which is beneficial for attracting students’ attention and decreasing their cognitive load. Especially for learning fractions, the content structure is complex and students need to conduct relational reasoning to understand. The structured presentation is helpful for enhancing relational reasoning about concepts by visually emphasizing the structure of the content (Kalra et al., 2020). This helps learners extract key information from static information and process it quickly (Tversky et al., 2008; Boucheix et al., 2013), enabling them to grasp the entire content (Gross and Harmon, 2009). Guided by a structured presentation, students can easily integrate a new topic with previous knowledge and comprehend the structure of a concept, which not only decreases students’ cognitive load but also increases their confidence about new teaching content (Bransford et al., 2000; Smith and Shimeld, 2014).

According to previous studies on the effects of segmenting and cueing, structured presentations also improve knowledge retention (Schneider et al., 2018; Rey et al., 2019). Previous research has proved that event-structured knowledge is understandable and easily recalled (Carter, 1994) because it reflects the logical relationships within teaching content and facilitates the identification and subsequent representation of the material’s structural organization (De Koning et al., 2009; Lee et al., 2018). Based on proven techniques (Boucheix and Guignard, 2005; De Koning et al., 2009; Boucheix et al., 2013; Richter et al., 2016), key information can be highlighted, and its relationships with relevant knowledge can be presented by using different signals to visualize organized knowledge elements, development logic, and structural hierarchy. Visualization of the knowledge structure can support learners in integrating the elements between and within representations into a coherent whole (Wouters et al., 2008; De Koning et al., 2010), which is beneficial for constructing schemata of the knowledge and committing them to memory.

### 2.3. Stepwise presentations add dynamic stimuli to attract students’ attention

Stepwise is regarded as a key dynamic cue for presenting teaching content. Dynamic cues are privileged by the human visual system (Wolfe and Horowitz, 2004). Because of their dynamic nature, these cues can present interactive relationships among knowledge elements in a way that static signals cannot. Particularly for abstract cognitive processes, dynamic signals can facilitate the externalization of cognitive processes better than verbal descriptions (Wouters et al., 2008). Combining visual animation with narration encourages students to process information at a deeper level than narration or on-screen text alone (Dunsworth and Atkinson, 2007). Thus, the stepwise presentation can guide students’ attention to dynamic elements by presenting different stimuli or posing different questions in each step. Under the guidance of the stepwise presentation, a new object, as the learning input can be quickly captured by students’ perceptual system (Chen et al., 2016; Lei et al., 2017), and meanwhile, students’ attention can be attracted to the location where the new objects occurred (Yantis and Jonides, 1996).

The stepwise presentation is also based on segmentation, which breaks the complex content into simple elements to decrease interactivity (Chen et al., 2016; Lee et al., 2018). As we all know, fraction concepts involve multiple elements that interact with each other, and presenting them all at once will increase students’ cognitive load (Mayer and Pilegard, 2014). Thus, during teaching practice, decomposing the fraction concept is a common approach to reduce the complexity. Segmentation, as the first step of the stepwise presentation, can break the fraction concept into simple components, each unit representing a natural number that the students learned before. Combined with oral guidance and coloring, the stepwise presentation can attract students’ attention to the key point step by step (Jamet et al., 2008). This can also provide scaffolding for students to build relationships between components. Therefore, stepwise presentations can help students construct knowledge actively.

## 3. Research questions and hypotheses

Existing studies have shown that structured presentations and stepwise presentations individually enhance students’ learning (Miao et al., 2000; Tso et al., 2011; Kalra et al., 2020), which play different roles in teaching guidance. The purpose of the structured presentation is to visualize the overall static structure of mathematics content itself, and stepwise, as a kind of dynamic teaching guidance technique, aims to strengthen the generating logic of mathematical structure. However, the application of combing structured presentations and stepwise presentations in fraction teaching still remains unexplored. Especially regarding the effect of integrating these two methods on students’ attention has received little attention from researchers. Therefore, in this study, we aimed to improve students’ learning efficiency by combining different visual presentation approaches, including structured presentations, and stepwise presentations, which we called structured stepwise presentations, to draw students’ attention. The research question asked whether students’ attention and learning performance are influenced by structured stepwise presentations of fraction concepts. Based on prior studies, the hypotheses in this study were as follows:

*H1*: Structured stepwise presentations can draw students’ attention during the learning of fractions.

*H2*: Structured stepwise presentations are advantageous for encouraging students to connect interactive elements between fraction concepts.

*H3*: Students in the group using structured stepwise presentations can learn fraction concepts more easily than other groups.

## 4. Methods

### 4.1. Participants

A total of 100 third-grade students from a primary school (44 female and 56 male), none of whom had studied fractions before, were selected from 10 classes. The age of these students is eight or nine. Considering that neither structured nor stepwise presentation of fractions is relatively rare in teaching practice, we only focused on three conditions in this experiment. Initially, the participants were randomly assigned to one of these three conditions: a structured and stepwise (SaS) group, *n* = 33; an unstructured and stepwise (UaS) group, *n* = 33; and a structured and not stepwise (SaN) group, *n* = 34. After the experiment, five participants were excluded because their eye-tracking rates were below 80%. Finally, 95 participants (SaS group *n* = 31, UaS group *n* = 32, SaN group *n* = 32) were included in the analysis. We examined these participants’ prior knowledge in the school before studying fractions. The test lasted 1 h and the total score was 100. The content was mainly related to the algebra knowledge that students have learned before, including multi-digit addition and subtraction, one-digit multiplication and division, the concept of decimals, the addition and subtraction of one-digit decimals, and the application of these concepts. After using analysis of variance (ANOVA), we found no significant differences among the three groups: *F* (2, 93) = 0.614, *p* = 0.544 > 0.05, which indicated the same level of mathematical ability of these students.

### 4.2. Instruments

**Figure 1:**
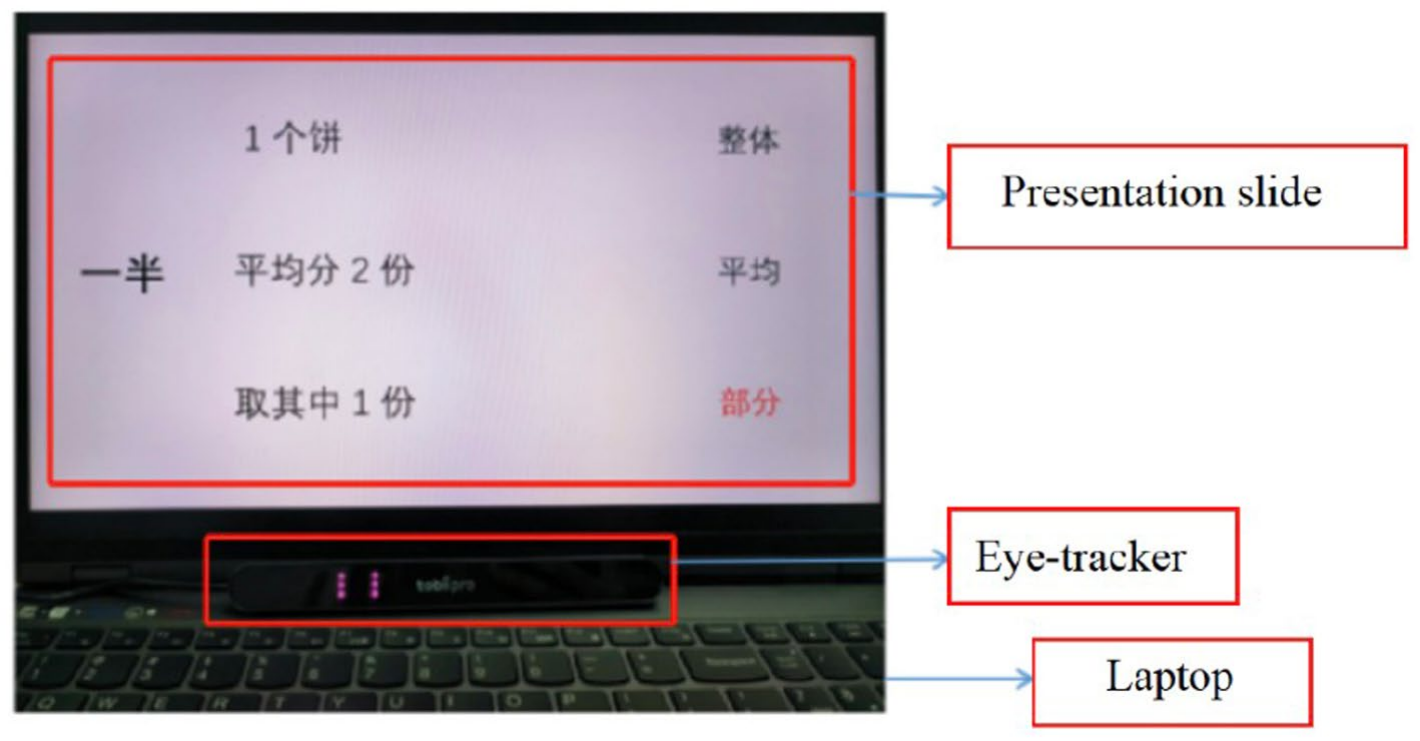
The device used in this study.

We used a Tobii Pro X-60 stationary eye tracker to record the participants’ eye-movement information. We installed it at the bottom of a 24-inch computer screen (see Figure 1), with a display resolution of 1,024 × 768 pixels, which we used to present different stimuli to the participants. We collected the participants’ binocular eye-movement data at a 60-Hz sample rate. Before starting the experiment, we calibrated the eye tracker for each participant using a nine-point calibration.

After the experiment, each participant in the three groups was given 15 min to finish the post-test in order to examine the learning effect. Five questions tested the participants’ understanding of the concept of fractions, and the total possible score was 30. Three of the questions tested the students’ recollection of the presented teaching content, including a realistic explanation of a fraction and its function in mathematics. The other two questions tested students’ simple relational reasoning about fractions.

### 4.3. Procedure

The two key variables in this experiment were *structured* and *stepwis*e. In the structured knowledge presentation, we used a row-and-column organization of two-dimensional space to establish corresponding relationships. For example, to present the fraction concept, we divided the presentation into three columns: the left column presented key information about a real-life scenario, the middle column presented the symbolic mathematical elements, and the right column presented the literal mathematical elements. The information relating to the numerator, score line, and denominator was presented in rows and arranged according to the corresponding relationship. Thus, structured connections between fraction concepts could be visualized through correlations between real-life information, mathematical symbols, and mathematical elements or through abstract correlations between the key elements of the fraction. Additionally, In the stepwise presentation, each page presented different visual stimuli, distinguished information with distinct colors, and posed different problems to encourage the students to think. The stepwise action was prompted by “clicking,” combined with oral guidance. Therefore, we assigned the participants to the three experimental groups based on the above variables, namely the SaS group, UaS group, and SaN group, as previously outlined. The presentation content, the teacher’s explanations, and the teaching method were the same for all three groups; only the PowerPoint (PPT) presentation modes differed.

In the SaS group, the teaching presentation was structured and stepwise. The presentation slides were synchronized with the teacher’s oral guidance, and each slide presented only one piece of crucial information, marked in red, which would turn black in the next step. We arranged the teaching content according to its structure. After all the slides had been presented, the students could see the structure of the entire content.

In the UaS group, the presentation was designed unstructured but stepwise with color. We arranged everything linearly in the order that it should be dealt with. The number of slides in the UaS group was the same as in the SaS group, but the arrangement of the PPT content differed.

In the SaN group, the presentation was structured, but there were no steps. The content was presented on one slide before the teacher gave oral guidance. Therefore, the number of slides was smaller than for the other groups.

**Figure 2:**
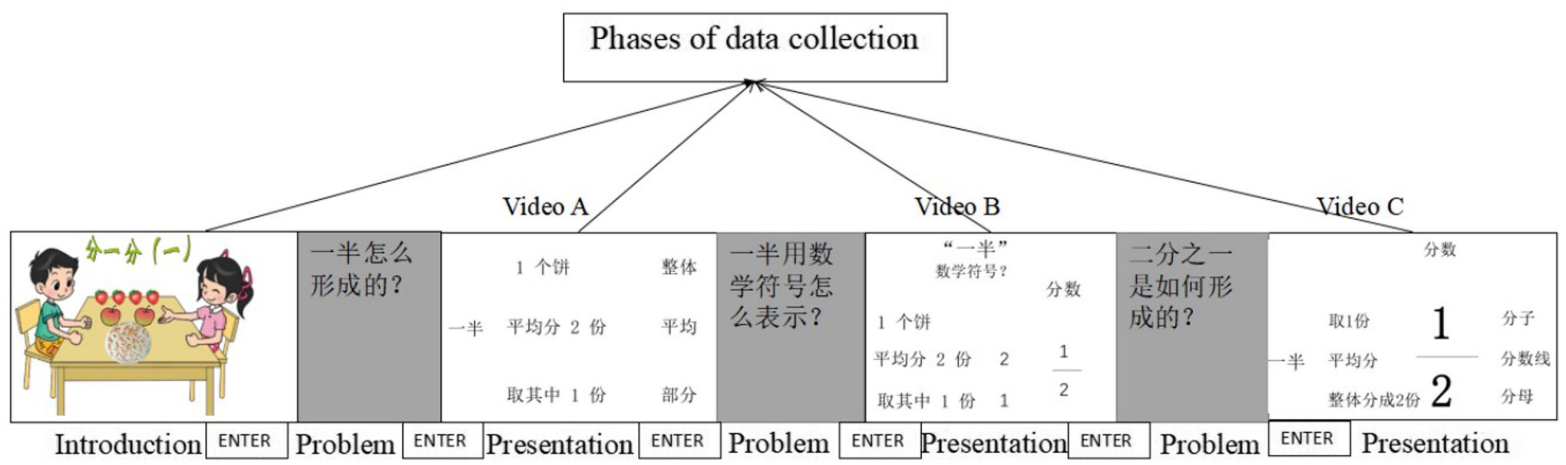
Flow diagram of the eye-tracking experiment (SaS example).

The flow diagram of the eye-tracking experiment using the SaS group as an example is shown in Figure 2. The teaching for each group consisted of four phases, introduction, and then presenting three videos (videos A–C). The introduction part was used to allow students to experience the concept of average by dividing apples in a real-life situation. After that, videos A–C were played consecutively at the same interval and each video was preceded by a guiding question. The total length of these three videos was 8 min and 15 s. For the SaS group and the UaS group, the numbers of slides in videos A, B, and C were 7, 6, and 11, respectively, but there was only one slide in each video for the SaN group. To measure the instantaneous effect of PPT design on students’ learning, all students were only given one chance to watch the videos and were not allowed to review them.

### 4.4. Data analysis

In this experiment, the independent variable was the technological presentation mode, and students’ eye movements and learning performance were the dependent variables. To measure students’ visual attention, we adopted the following eye-movement indicators: the first and total durations of fixation in the areas of interest (AOIs) and the regression time, which referred to the total fixation duration in which students related the current AOI to another AOI (Rayner et al., 2009; Jian et al., 2019).

**Figure 3:**
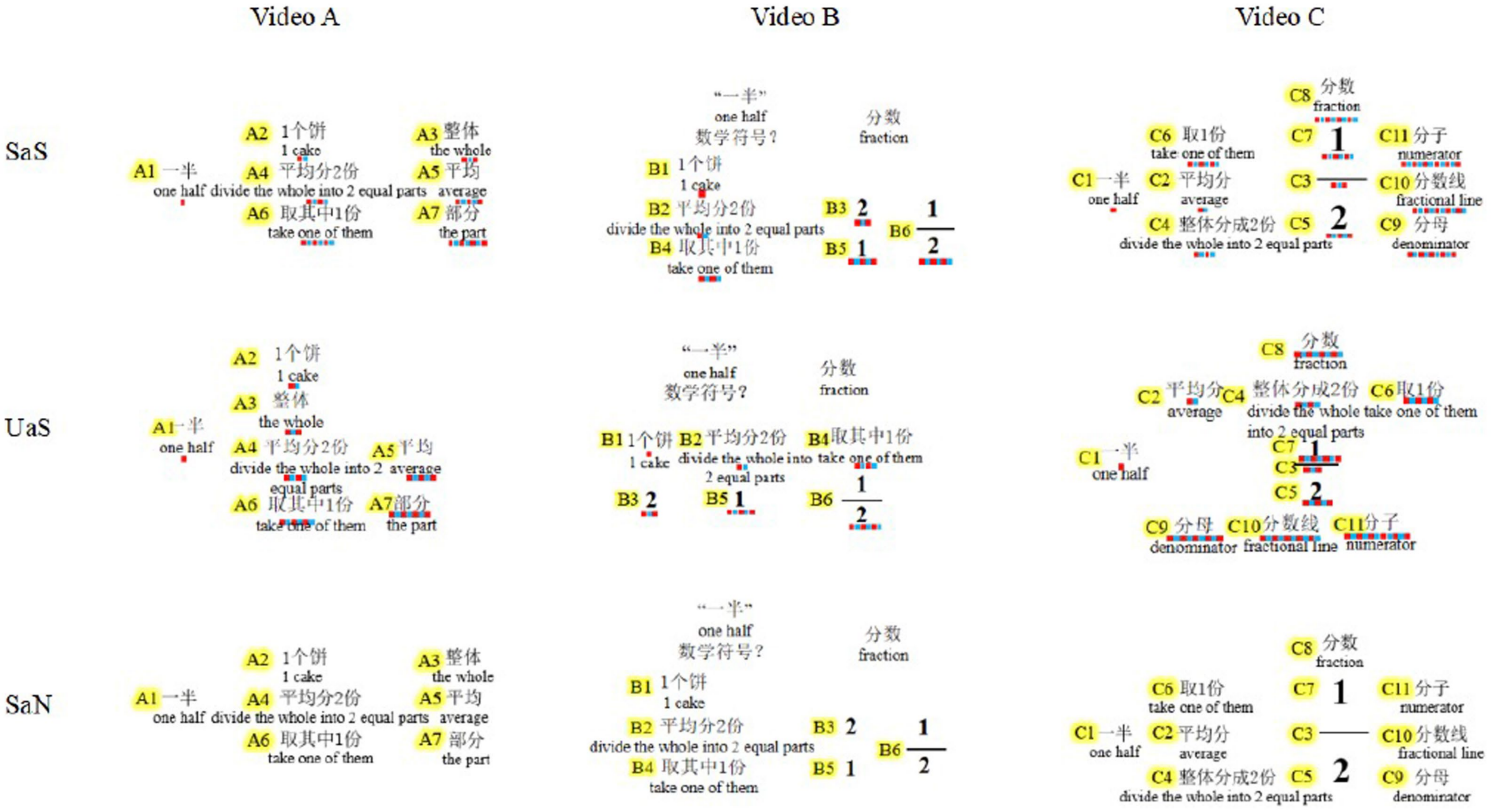
AOI Labels representing teaching content in each video for three groups. Color marking below each AOI for the SaS group and UaS group in the figure represents the sequence of AOI steps; there is no color marking in the SaN group, as the whole contents are presented at once with no steps.

Before analyzing the eye movements, we first defined the AOIs in each video for the three groups, as shown in Figure 3. Each AOI represented a keyword or key sentence in the teaching content. There were 24 AOIs for each group, which were marked AOI(A1), …, AOI(A7); AOI(B1), …, AOI(B6); and AOI(C1), …, AOI(C11). The capital letter in parentheses represents the name of the video (A, B, or C), and the number represents the serial number of the AOI in that video. Although there shows similar presenting content in some AOIs, it conveyed different meanings during teaching. The first video was used to introduce the real-life information of one half; the second video was used to guide students to extract mathematical information from life language, and convert them into mathematical symbols; the third video was used to connect real-life information and fraction elements, in order to foster students’ deep understanding of the fraction concept.

In other words, there are diverse ways to express the fraction concept by relating various elements from different perspectives. In this study, the related elements were formed by two kinds of AOIs: one used literal mathematical language, and the other consisted of real-life information or symbolic mathematical language. Taking video A as an example, the fraction concept had three mathematical components: the whole AOI(A3), the average AOI(A5), and the part AOI(A7), which, respectively, connected to the students’ own life experience, one piece of cake (AOI(A2)) divided into two equal parts (AOI(A4)), and one part removed (AOI(A6)). Thus, we obtained three related elements: AOI(A2–A3), AOI(A4–A5), and AOI(A6–A7). Finally, for three videos, there were 11 related elements, including AOI(A2–A3), AOI(A4–A5), AOI(A6–A7), AOI(B2–B3), AOI(B4–B5), AOI(C2–C3), AOI(C4–C5), AOI(C6–C7), AOI(C3–C10), AOI(C5–C9), and AOI(C7–C11). To analyze whether students made connections between two related AOIs, we used the regression time to characterize the duration of students’ looking back to the former AOI when the teaching guided the students to the later AOI. For example, the fixation duration for AOI(A2–A3) represented the amount of time that students’ attention dwelled on the former AOI(A2) when the teaching pointed to the later AOI(A3). The specific calculation approach was as follows: when the first fixation time on AOI(A2) preceded fixation on AOI (A3), the regression time for AOI (A2–A3) was the total fixation duration on AOI(A2) minus the first fixation duration on AOI(A2). When the first fixation time on AOI(A2) lagged behind that on AOI(A3), we used the total fixation duration on AOI(A2) to depict the regression time on AOI(A2–A3). Thus, regression time could be used to reveal students’ ability to understand and integrate information (Schotter et al., 2014).

We gathered data about students’ learning performance from the test that followed the experiment. All students were given as much time as needed to answer the questions and finish the test. The reliability values of the test scores for the three groups were 0.925 (SaS group), 0.890 (UaS group), and 0.917 (SaN group), respectively. We analyzed the first fixation duration, the total fixation duration, and the regression time of the students’ eye movements across AOIs during learning. We used IBM® SPSS® 27.0 software to conduct the quantitative analysis. The significance level α was set at 0.05, and we used a one-way ANOVA to check for differences between the three groups.

## 5. Results

### 5.1. Significant differences in the attention of the three groups

The statistical data for the first and total fixation durations of eye movement confirmed that the structured stepwise presentation had a strong attention-guiding effect, which was consistent with H1.

Table 1 shows the students’ first fixation duration for each AOI in the three groups. A one-way ANOVA showed that the first fixation duration of the three groups differed significantly. Considering that several independent statistical tests were performed simultaneously, we conducted the Bonferroni correction by taking the alpha value for each comparison equal to 0.05/24 (0.002). The results showed significant differences for the following 12 AOIs: AOI(A1), *F*(2, 93) = 13.691, *p* < 0.002, *η*^2^ = 0.229; AOI(A2), *F*(2, 93) = 11.698, *p* < 0.002, *η*^2^ = 0.203; AOI(A3), *F*(2, 93) = 13.405, *p* < 0.002, *η*^2^ = 0.226; AOI(A6), *F*(2, 93) = 6.443, *p* = 0.002, *η*^2^ = 0.123; AOI(C1), *F*(2, 93) = 18.207, *p* < 0.002, *η*^2^ = 0.284; AOI(C2), *F*(2, 93) = 10.472, *p* < 0.002, *η*^2^ = 0.185; AOI(C5), *F*(2, 93) = 9.498, *p* < 0.002, *η*^2^ = 0.171; AOI(C6), *F*(2, 93) = 7.617, *p* = 0.001, *η*^2^ = 0.142; AOI(C8), *F*(2, 93) = 7.868, *p* = 0.001, *η*^2^ = 0.146; AOI(C9), *F*(2, 93) = 11.661, *p* < 0.002, *η*^2^ = 0.202; AOI(C10), *F*(2, 93) = 8.690, *p* < 0.002, *η*^2^ = 0.159; and AOI(C11), *F*(2, 93) = 16.686, *p* < 0.002, *η*^2^ = 0.266. Considering the equal variances assumed, we used the Least Significant Difference test (LSD) for multiple comparisons to test which group differs, and the results were marked with subscript letters in Table 1 at *p* < 0.002. The first fixation duration of the students in the SaS group was considerably longer than that of the other groups. The next longest duration was in the UaS group, and the first fixation time of the SaN group was the lowest among the three groups. Additionally, the number of AOIs with significant differences in the first fixation duration between the SaS group and the SaN group was higher than the number of AOIs with differences between other groups. Furthermore, it was hard to find significant differences between groups in video B. Therefore, in the SaS group, the students were stimulated by structured stepwise presentations, and their first attention was better than that of the other two groups.

**Table 1:** One-way ANOVA of the first fixation duration for AOIs (time in milliseconds).

	SaS group		UaS group		SaN group		*F*	*p*	*η*^2^
	*M*	SD	*M*	SD	*M*	SD			
Video A									
A1	0.818a	0.510	0.290b	0.530	0.335b	0.220	13.691	<0.001	0.229
A2	0.787a	0.532	0.635a	0.641	0.206b	0.213	11.698	<0.001	0.203
A3	0.612a	0.384	0.269b	0.231	0.265b	0.280	13.405	<0.001	0.226
A4	0.751a	0.438	0.749a	0.992	0.414a	0.331	2.783	0.067	0.057
A5	0.540a,b	0.416	0.586a	0.612	0.224b	0.282	5.905	0.004	0.114
A6	0.651a	0.442	0.560a,b	0.453	0.294b	0.327	6.443	0.002	0.123
A7	0.439a	0.261	0.431a	0.521	0.262a	0.309	2.185	0.118	0.045
Video B									
B1	0.359a	0.201	0.389a	0.292	0.353a	0.327	0.156	0.856	0.003
B2	0.685a	0.660	0.525a	0.485	0.390a	0.502	2.244	0.112	0.047
B3	0.324a	0.380	0.258a	0.364	0.091a	0.183	4.408	0.015	0.087
B4	0.692a	0.836	0.666a	0.734	0.353a	0.654	2.040	0.136	0.042
B5	0.314a	0.312	0.277a	0.458	0.056a	0.142	5.670	0.005	0.110
B6	0.737a	0.604	0.603a	0.742	0.759a	0.885	0.401	0.671	0.009
Video C									
C1	0.448a	0.443	0.109b	0.201	0.048b	0.084	18.207	<0.001	0.284
C2	0.662a	0.596	0.579a	0.657	0.095b	0.261	10.472	<0.001	0.185
C3	0.599a	0.682	0.464a,b	0.427	0.173b	0.346	5.915	0.004	0.114
C4	0.938a	0.722	0.696a,b	1.093	0.122b	0.242	9.378	<0.001	0.169
C5	0.433a,b	0.541	0.688a	0.751	0.097b	0.168	9.498	<0.001	0.171
C6	0.530a	0.485	0.217a,b	0.709	0.042b	0.133	7.617	0.001	0.142
C7	0.566a	0.784	0.570a	0.919	0.157a	0.528	3.103	0.050	0.063
C8	0.549a	0.787	0.256a,b	0.248	0.063b	0.219	7.868	0.001	0.146
C9	0.622a	0.497	0.403a,b	0.482	0.118b	0.201	11.661	<0.001	0.202
C10	0.823a	0.709	0.514a,b	0.526	0.249b	0.350	8.690	<0.001	0.159
C11	0.651a	0.492	0.279b	0.421	0.087b	0.217	16.686	<0.001	0.266

Values with different subscript letters in a row differ significantly at *p* < 0.002.

Table 2 shows the total fixation duration of the students for each AOI. After the Bonferroni correction, there showed significant differences among the three groups for 19 AOIs at *p <* 0.002: AOI(A1), AOI(A2), AOI(A3), AOI(A4), AOI(A5), AOI(A6), AOI(A7), AOI(B2), AOI(C1), AOI(C2), AOI(C3), AOI(C4), AOI(C5), AOI(C6), AOI(C7), AOI(C8), AOI(C9), AOI(C10), and AOI(C11). There were no significant differences for the following AOIs: AOI(B1), AOI(B3), AOI(B4), AOI(B5), AOI(B6), and AOI(C1). We further performed the LSD test for multiple comparisons, as shown in Table 2 with subscript letters. There were significant differences in the total fixation duration of most AOIs between any two groups, especially between the SaS group and the SaN group. Specifically, the total fixation duration for most AOIs of the SaS group was significantly longer than that of the other groups, and the total fixation duration for all AOIs of the SaN group was the lowest among the three groups. In other words, the effect of stepwise presentations was higher than the effect of structured presentations. Even for the content with a low cognitive load, the stepwise presentation also worked to attract students’ visual attention.

**Table 2:** One-way ANOVA of the total fixation duration for AOIs (time in milliseconds).

	SaS group		UaS group		SaN group		*F*	*p*	*η*^2^
	*M*	SD	*M*	SD	*M*	SD			
Video A									
A1	4.364a	2.614	0.535b	0.754	0.766b	0.428	58.183	<0.001	0.558
A2	1.471a	0.817	1.049a	0.804	0.31b	0.306	23.391	<0.001	0.337
A3	1.616a	0.892	0.658b	0.749	0.494b	0.471	21.999	<0.001	0.324
A4	3.151a	1.136	1.893b	1.238	1.017c	0.938	29.316	<0.001	0.389
A5	1.777a	1.142	1.252a,b	1.128	0.488b	0.705	12.992	<0.001	0.220
A6	2.465a	1.046	1.372b	1.046	0.613c	0.717	30.320	<0.001	0.397
A7	2.067a	1.874	1.591a,b	1.626	0.612b	1.137	7.043	0.001	0.133
Video B									
B1	1.143a	0.773	1.171a	0.946	0.780a	0.727	2.051	0.134	0.043
B2	2.119a	1.161	1.347a,b	1.238	0.696b	0.74	14.006	<0.001	0.233
B3	0.744a	0.832	0.393a	0.645	0.217a	0.66	4.408	0.015	0.087
B4	1.779a	1.412	1.248a	1.26	0.836a	0.992	4.636	0.012	0.092
B5	0.672a	0.799	0.500a,b	0.822	0.091b	0.277	6.113	0.003	0.117
B6	2.679a	2.139	1.467a	1.899	1.508a	1.405	4.401	0.015	0.087
Video C									
C1	0.771a	0.701	0.160b	0.368	0.041b	0.078	23.121	<0.001	0.335
C2	1.465a	0.753	0.939a	0.849	0.139b	0.372	29.655	<0.001	0.392
C3	1.881a	1.324	0.908b	0.859	0.201b	0.367	25.848	<0.001	0.360
C4	2.239a	0.991	1.171b	1.294	0.189c	0.388	35.430	<0.001	0.435
C5	0.799a	0.878	0.889a	0.868	0.120b	0.207	10.854	<0.001	0.191
C6	1.462a	1.017	0.267b	0.729	0.042b	0.133	34.865	<0.001	0.431
C7	1.593a	1.027	0.958a	1.029	0.217b	0.691	17.351	<0.001	0.274
C8	1.237a	1.208	0.481b	0.609	0.063b	0.219	17.993	<0.001	0.281
C9	1.795a	0.941	0.799b	0.806	0.213b	0.460	34.741	<0.001	0.430
C10	1.775a	1.005	0.892b	0.886	0.388b	0.598	21.672	<0.001	0.320
C11	1.903a	1.230	0.338b	0.573	0.088b	0.217	48.906	<0.001	0.515

Values with different subscript letters in a row differ significantly at *p* < 0.002.

In sum, the structured stepwise presentation efficiently attracted the students’ attention in fraction learning, thereby verifying H1 to a certain extent. There could be some possible reasons for no significant differences in the first fixation durations and total fixation durations of some AOIs among the three groups. Firstly, the average time of the first fixation duration was too short (less than 1 s), which may cause no statistically significant difference between the three groups. This also resulted in more AOIs with no significant difference in first fixation duration than that in total fixation duration. Secondly, most AOIs with no significant difference were from video B, which involves fewer abstract concepts and a lower intrinsic cognitive load required than the other two videos. In another word, this indicates that the structured stepwise presentation is particularly effective for the contents with high intrinsic cognitive load.

### 5.2. Significant differences in connecting corresponding AOIs

In three videos, we designed 11 indices to show the structured connections between corresponding AOIs in the presentation and used the regression time to evaluate the significance of attention across the two corresponding AOIs. The one-way ANOVA results showed significant differences between the three groups for the following seven related elements, as shown in Table 3, and the alpha value was set at 0.004 (0.05/11): AOI(B2–B3), *F*(2, 93) = 11.094, *p* < 0.004, *η*^2^ = 0.194; AOI(B4–B5), *F*(2, 93) = 9.531, *p* < 0.004, *η*^2^ = 0.172; AOI(C2–C3), *F*(2, 93) = 12.665, *p* < 0.004, *η*^2^ = 0.216; AOI(C4–C5), *F*(2, 93) = 12.949, *p* < 0.004, *η*^2^ = 0.220; AOI(C6–C7), *F*(2, 93) = 6.961, *p* = 0.002 < 0.004, *η*^2^ = 0.131; AOI(C5–C9), *F*(2, 93) = 7.241, *p* < 0.004, *η*^2^ = 0.136; and AOI(C7–C11), *F*(2, 93) = 10.622, *p* < 0.004, *η*^2^ = 0.188. After the LSD test (see Table 3), the results showed significant differences in the regression time for more than half of the related AOIs between the SaS group and the UaS group or the SaN group. Specifically, the regression time of the SaS group was longer than that of the UaS group or the SaN group. That is to say, the effect of the structured presentations on the regression time is almost as significant as the effect of the stepwise presentations. Therefore, the structured stepwise presentations were helpful in guiding students to establish relationships between the corresponding elements. Furthermore, most of the regression time differences were found in videos B and C, in which students were experiencing the challenging process of expressing “half” in mathematical language. This indicates that students can make connections between symbolic mathematical language and real-life information or literal mathematical language with step oral guidance. In comparison, video A was mainly used to recall prior knowledge and introduce the concepts, namely whole, average, and part, which not involving different languages for students to relate. Thus, the differences in the regression time between the three groups were not significant as the differences in the fixation duration between them.

**Table 3:** One-way ANOVA of the regression time between corresponding AOIs (time in milliseconds).

	SaS group		UaS group		SaN group		*F*	*p*	*η*^2^
	*M*	SD	*M*	SD	*M*	SD			
Video A									
A2–A3	0.475a	0.541	0.198a	0.287	0.383a	0.477	3.165	0.047	0.064
A4–A5	1.137a	0.906	1.358a	1.116	0.633a	0.740	5.075	0.008	0.099
A6–A7	0.973a	1.698	0.703a	0.734	0.620a	0.940	0.752	0.474	0.016
Video B									
B2–B3	0.702a	0.654	0.912a	1.027	0.102b	0.203	11.094	<0.001	0.194
B4–B5	0.138a	0.231	0.557a	0.749	0.081a	0.238	9.531	<0.001	0.172
Video C									
C2–C3	0.902a	0.756	0.481a,b	0.554	0.171b	0.363	12.665	<0.001	0.216
C4–C5	0.588a	0.652	0.156a	0.356	0.048a	0.218	12.949	<0.001	0.220
C6–C7	0.174a	0.309	0.027a	0.109	0.013a	0.042	6.961	0.002	0.131
C3–C10	0.083a	0.322	0.015a	0.050	0.019a	0.110	1.165	0.316	0.025
C5–C9	0.211a	0.287	0.104a,b	0.202	0.016b	0.052	7.241	0.001	0.136
C7–C11	0.625a	0.796	0.273a,b	0.432	0.021b	0.092	10.622	<0.001	0.188

Values with different subscript letters in a row differ significantly at *p* < 0.004.

### 5.3. Structured stepwise presentation achieved better learning performance

The total test score for students’ learning performance was 30. All the students’ answers were reliable and valid. Unfortunately, the one-way ANOVA results showed no statistically significant differences among the three groups, *F*(2, 92) = 2.939, *p* = 0.058, *η*^2^ = 0.06. There could be other important factors that influence students’ learning performance, such as emotions, metacognitive abilities, and personality traits. We have to consider the delayed effect of the concentrating process on the final learning achievement. Despite this fact, the average score of students in the SaS group (*M* = 23.566, SD = 6.612) was still higher than that in the UaS group (*M* = 19.606, SD = 7.697), and the SaN group (*M* = 19.875, SD = 7.469). But the score for the UaS group was close to the score for the SaN group. In another word, the students in the SaS group who paid more attention to key information and related elements achieved better learning performance to some extent. This indicates that the structured stepwise presentation could have a beneficial effect on the students’ fraction learning.

## 6. Discussion and conclusion

In this study, we investigated the effect of structured stepwise presentations on students’ learning of fractions using eye-tracking technology. The results showed that the structured stepwise presentations played an important synergistic role in directing students’ attention and promoting learning performance.

### 6.1. Dynamic stimulation to guide and strengthen students’ attention

The results of this study showed that the mean fixation duration of students in the SaS and UaS groups, who learned with stepwise presentations, was greater than that of the students in the SaN group. In other words, more attention was allocated to the AOIs in the stepwise presentation groups. These results confirmed that stepwise presentations as dynamic signals favorably guided students’ attention, which was consistent with the existing research (De Koning et al., 2009; Tso et al., 2011).

In fact, the stepwise effect was enhanced by dynamic stimulation. The stepwise format intensified the dynamic nature of the presentation, which stimulated students’ attention. Since a new object was generated in each step, the learning input could be sequentially captured by students’ attention and effectively prevent them from being distracted by external factors (Boucheix and Guignard, 2005; Wouters et al., 2008; Lowe and Schnotz, 2014). Taking the experimental group’s teaching as an example, since the teacher presented each slide in sequence, key information was shown to the students gradually, which provided dynamic signals for the students and functioned as a stimulus to attract the students’ attention to the cues. Thus, in the SaS and UaS groups, the students’ first fixation duration on each AOI was longer than that of students in the SaN group. As prior studies have shown, dynamic signaling not only captures students’ attention but also focuses their attention on key processes and prevents distraction (Wouters et al., 2008; Boucheix et al., 2013).

Stepwise presentations can effectively support the teaching of complex content and provide scaffolding for students’ knowledge construction by segmenting the content into several related parts (Lee et al., 2018). In the SaS group, the teaching presentation showed multiple representations of the fraction concept. Each scaffolding sub-concept involved key elements for learning fractions, including real-life applications, literal mathematical language, and symbolic mathematical language, which were presented in videos B and C. As Table 3 showed, the effect of relating the corresponding elements was better in the SaS group than that in the SaN group. Thus, stepwise presentations can help students to identify the connection between related information blocks, which provided a constructive learning process that transformed real-life information into mathematical language and presented an overall concept-forming process (Chen et al., 2016; Rey et al., 2019). The eye-tracking data in this study suggested that the stepwise presentations were effective to direct students’ attention. In fact, the stepwise effect can release more cognitive resources for students to construct knowledge (Rey et al., 2019). In each stepwise connecting block, the teacher provided a construction cue using dynamic stimuli. Thus, differences between the upper and lower blocks could be easily observed by the students. Therefore, stepwise presentations for abstract information can be understood in terms of effective connections between accessible sub-elements, and problems in the learning of fractions, such as situation distortion, monotonous content, and lack of a constructive process, can be solved.

### 6.2. Structured presentations are conducive to the formation of concepts

This study showed that structured presentations better encourage students to make connections between related elements. A structured presentation visualizing the potential conceptual relationships, especially in videos B and C, had a significant effect on students’ attention to connect related elements in the SaS group, as shown in Table 3. It revealed that students’ attention was attracted by structured cueing, which helped them integrate related elements (Schotter et al., 2014; Eskenazi and Folk, 2017). The results confirm the effectiveness of the signaling principle for visualizing semantic and grammatical rules. Structured presentations convert seeing into understanding, allowing semantics and grammar to be distinguished by visualizing the relationships between learning concepts (Miao et al., 2000; De Koning et al., 2010). Thus, in the SaS group, students easily identified related elements and had a stronger disposition to fixate on them than in the other groups. The results confirmed that effective visual signals enable connections to be made and knowledge to be clearly visualized, reducing the burden of interpretation (De Koning et al., 2010).

A structured presentation also guides students to form concepts step-by-step and supports them in a concept-forming process that includes exploration, reflection, and discovery. Visual structured knowledge representations not only help students internalize and reflect on their knowledge but also support negotiation and exploration (Miao et al., 2000). In this study, the SaS group had better attention and more effectively made connections between corresponding AOIs, indicating that these students experienced a fruitful learning process. This aligns with research showing that regression time is informational and beneficial for students’ understanding and connection-making (Rayner et al., 2009; Schotter et al., 2014; Jian et al., 2019). Furthermore, based on the cueing principle, according to presented visual stimuli, such as color, information blocks, and identifiers, students constantly adjust the direction of their attention to understand the content and reflect on it, which is beneficial for promoting self-regulated learning (Ferrara and Butcher, 2011). Therefore, students’ active construction of knowledge can be supported by structured presentations.

In addition, structured presentations give students a deep impression of their knowledge. In this study, regression time reflected the cognitive process of integrating, rather than simply extracting information (Rayner et al., 2009; Jian et al., 2019), and the regression for relevant information was applied more often by high-level learners (Mason et al., 2013). In this study, compared to other groups, the students in the SaS group obtained higher scores on the test that included several recall questions, and this result indicated that structured knowledge is well remembered (Carter and Kathy, 1994). The prior study suggested that visual reinforcement stimulation is more conducive to processing text and symbols than language stimulation during the process of short-term memory formation (Liang, 2014). Structured presentations, by visually presenting the structure of knowledge and imaginable characteristics, help students form refined short-term memory and knowledge schemata, which are easily extracted, retained, and recalled. Dedicated short-term memory supports students’ effective long-term memory storage, which can provide the basis for forming strong knowledge structures. Therefore, structured knowledge presentations help students realize the fine processing of knowledge and the effective construction of knowledge schemata under conditions of limited working memory.

### 6.3. Conclusion

In this study, we confirmed that the structured stepwise presentation drew students’ attention, guided them to understand the cognitive process of learning about fractions, and promoted their learning performance. Furthermore, the results showed that stepwise presentations worked better than structured presentations to attract students’ visual attention. Structured presentations are effective for complex concepts with high-element interactivity, but are not as remarkable as for contents with low cognitive load. Furthermore, stepwise presentations can accelerate the effect of structured presentations, which are adaptive to the content with different cognitive levels. The findings of this study reveal that a good presentation is vital for helping students construct knowledge and guiding them to deeply understand new content. In order to achieve high-quality fraction teaching, we suggested that teachers should combine structured presentations and stepwise presentations together to provide dynamic cues in a well-organized way, and further clarify the relationships between different to support students in forming clear knowledge schemata.

In sum, we examined the rationale of structured stepwise presentation based on the principle of segmenting and cueing. However, there are some limitations in this study. We used only eye-movement indicators to analyze the students’ attention. Future studies could find other ways to evaluate students’ attention and investigate whether an extraneous cognitive load can be decreased by structured stepwise presentations. In terms of the control of research variables, it is necessary to investigate the learning characteristics of each research object in the future and explore which kind of students is more effective under the guidance of structured stepwise presentations. Regarding the learning test, this study examined students’ learning performance based on memory understanding of fraction concepts. Whether structured stepwise presentations can be used to solve more complex cognitive problems and promote students’ advanced thinking should be examined in future research.

## Data availability statement

The raw data supporting the conclusions of this article will be made available by the authors, without undue reservation.

## Author contributions

XS designed and carried out the experiment. XS and RL analyzed the data and wrote the manuscript text. YL provided ideas for this manuscript. All authors contributed to the article and approved the submitted version.

## Funding

This research was supported by the Humanities and Social Science Foundation of Ministry of Education of China (22XJA880009), by the Science and Technology Foundation Project of Shaanxi (2022QFY01-05).

## Conflict of interest

The authors declare that the research was conducted in the absence of any commercial or financial relationships that could be construed as a potential conflict of interest.

## Publisher’s note

All claims expressed in this article are solely those of the authors and do not necessarily represent those of their affiliated organizations, or those of the publisher, the editors and the reviewers. Any product that may be evaluated in this article, or claim that may be made by its manufacturer, is not guaranteed or endorsed by the publisher.

## References

[ref1] Boucheix J. M. Guignard H. (2005). What animated illustrations can improve technical document comprehension in young students? Format, signaling, and control of the presentation. Eur. J. Psychol. Educ. 20, 369–388. doi: 10.1007/BF03173563

[ref2] Boucheix J. M. Lowe R. K. (2010). An eye tracking comparison of external pointing cues and internal continuous cues in learning with complex animations. Learn. Instr. 20, 123–135. doi: 10.1016/j.learninstruc.2009.02.015

[ref3] Boucheix J. M. Lowe R. K. Putri D. K. Groff J. (2013). Cueing animations: dynamic signaling aids information extraction and comprehension. Learn. Instr. 25, 71–84. doi: 10.1016/j.learninstruc.2012.11.005

[ref4] Bransford J. Brophy S. Williams S. (2000). When computer technologies meet the learning sciences: issues and opportunities. J. Appl. Dev. Psychol. 21, 59–84. doi: 10.1016/S0193-3973(99)00051-9

[ref5] Carter K. (1994). Preservice teachers’ well-remembered events and the acquisition of event-structured knowledge. J. Curric. Stud. 26, 235–252. doi: 10.1080/0022027940260301

[ref6] Chen M. J. Lee C. Y. Lei K. H. Yang C. Y. (2016). The effects of using stepwise attention-guiding multimedia instruction to learn properties of tangents of circles. Taiwan J. Math. Educ. 3, 1–30. doi: 10.6278/tjme.20161005.001

[ref7] Cierniak G. Scheiter K. Gerjets P. (2009). Explaining the split-attention effect: is the reduction of extraneous cognitive load accompanied by an increase in germane cognitive load? Comput. Hum. Behav. 25, 315–324. doi: 10.1016/j.chb.2008.12.020

[ref8] De Koning B. B. Tabbers H. K. Rikers R. M. Paas F. (2007). Attention cueing as a means to enhance learning from an animation. Appl. Cogn. Psychol. 21, 731–746. doi: 10.1002/acp.1346

[ref9] De Koning B. B. Tabbers H. K. Rikers R. M. Paas F. (2009). Towards a framework for attention cueing in instructional animations: guidelines for research and design. Educ. Psychol. Rev. 21, 113–140. doi: 10.1007/s10648-009-9098-7

[ref10] De Koning B. B. Tabbers H. K. Rikers R. M. Paas F. (2010). Attention guidance in learning from a complex animation: seeing is understanding? Learn. Instr. 20, 111–122. doi: 10.1016/j.learninstruc.2009.02.010

[ref11] Doolittle P. E. Altstaedter L. L. (2009). The effect of working memory capacity on multimedia learning: does attentional control result in improved performance? J. Res. Innov. Teach. 2, 7–25.

[ref12] Dunsworth Q. Atkinson R. K. (2007). Fostering multimedia learning of science: exploring the role of an animated agent's image. Comput. Educ. 49, 677–690. doi: 10.1016/j.compedu.2005.11.010

[ref13] Eskenazi M. A. Folk J. R. (2017). Regressions during reading: the cost depends on the cause. Psychon. Bull. Rev. 24, 1211–1216. doi: 10.3758/s13423-016-1200-9, PMID: 27873185

[ref14] Ferrara L. Butcher K. R.. (2011). Visualizing feedback: using graphical cues to promote self-regulated learning. In: *Proceedings of the thirty-third annual conference of the cognitive science society* No. 30, p. 33.

[ref15] Gross A. G. Harmon J. E. (2009). The structure of power point presentations: the art of grasping things whole. IEEE Trans. Prof. Commun. 52, 121–137. doi: 10.1109/TPC.2009.2020889

[ref16] Jamet E. (2014). An eye-tracking study of cueing effects in multimedia learning. Comput. Hum. Behav. 32, 47–53. doi: 10.1016/j.chb.2013.11.013

[ref17] Jamet E. Gavota M. Quaireau C. (2008). Attention guiding in multimedia learning. Learn. Instr. 18, 135–145. doi: 10.1016/j.learninstruc.2007.01.011

[ref18] Jian Y. C. Su J. H. Hsiao Y. R. (2019). Differentiated processing strategies for science reading among sixth-grade students: exploration of eye movements using cluster analysis. Comput. Educ. 142:103652. doi: 10.1016/j.compedu.2019.103652

[ref19] Kalra P. B. Hubbard E. M. Matthews P. G. (2020). Taking the relational structure of fractions seriously: Relational reasoning predicts fraction knowledge in elementary school children. Contemp. Educ. Psychol. 62:101896. doi: 10.1016/j.cedpsych.2020.101896 32831458 PMC7442207

[ref20] Kurby C. A. Zacks J. M. (2008). Segmentation in the perception and memory of events. Trends Cogn. Sci. 12, 72–79. doi: 10.1016/j.tics.2007.11.004, PMID: 18178125 PMC2263140

[ref21] Lee C. Y. Lei K. H. Chen M. J. Tso T. Y. Chen I. P. (2018). Enhancing understanding through the use of structured representations. Eurasia journal of mathematics science and technology education, 14(5), 1875-1886. Contemp. Educ. Psychol. 14:101896. doi: 10.29333/ejmste/85424

[ref22] Lei K. H. Chen M. J. Lee C. Y. Tso T. Y. Lin S. L. (2017). Multimedia instruction presented by integrated context to enhance understanding of compass-and-straightedge construction. Eur. J. Math. Sci. Technol. Educ. 13, 3735–3752. doi: 10.12973/eurasia.2017.00756a

[ref23] Liang N. J. (2014). Contemporary cognitive psychology. Shanghai: Shanghai Education Press.

[ref24] Lin F. L. Huang M. F. Leu Y. C. (1996). A developmental study on learning and teaching of beginning fractions. Chin. J. Sci. Educ. 4, 161–196.

[ref25] Lortie-Forgues H. Tian J. Siegler R. S. (2015). Why is learning fraction and decimal arithmetic so difficult? Dev. Rev. 38, 201–221. doi: 10.1016/j.dr.2015.07.008

[ref27] Lowe R. K. Schnotz W. (2014). “Animation principles in multimedia learning,” in Cambridge handbooks in psychology. The Cambridge handbook of multimedia learning. ed. Mayer R. E. (New York: Cambridge University Press), 513–546.

[ref28] Mason L. Tornatora M. C. Pluchino P. (2013). Do fourth graders integrate text and picture in processing and learning from an illustrated science text? Evidence from eye-movement patterns. Comput. Educ. 60, 95–109. doi: 10.1016/j.compedu.2012.07.011

[ref29] Mautone P. D. Mayer R. E. (2001). Signaling as a cognitive guide in multimedia learning. J. Educ. Psychol. 93, 377–389. doi: 10.1037/0022-0663.93.2.377

[ref30] Mayer R. E. (2014). “Principles for managing essential processing in multimedia learning: segmenting, pretraining, and modality principles” in Cambridge handbooks in psychology. The Cambridge handbook of multimedia learning. ed. Mayer R. E. (New York, NY: Cambridge University Press), 345–367.

[ref31] Mayer R. E. Pilegard C. (2014). “Principles for managing essential processing in multimedia learning: segmenting, pre-training, and modality principles” in Cambridge handbooks in psychology. The Cambridge handbook of multimedia learning. ed. Mayer R. E. (New York: Cambridge University Press), 316–344.

[ref32] Miao Y. Holst S. Holmer T. Fleschutz J. Zentel P.. (2000). *An activity-oriented approach to visually structured knowledge representation for problem-based learning in virtual learning environments*. International Conference on the Design of Cooperative Systems, pp. 303–318

[ref33] Mitchell C. E. Miller L. D. (2010). Tech prep academics: using real life connections to develop scientific and mathematical literacy. Sch. Sci. Math. 95, 417–422. doi: 10.1111/j.1949-8594.1995.tb10195.x

[ref34] Obersteiner A. Hoof J. V. Verschaffel L. Dooren W. V. (2015). Who can escape the natural number bias in rational number tasks? A study involving students and experts. Br. J. Psychol. 107, 537–555. doi: 10.1111/bjop.12161, PMID: 26566736

[ref35] Ozcelik E. Arslan-Ari I. Cagiltay K. (2010). Why does signaling enhance multimedia learning? Evidence from eye movements. Comput. Hum. Behav. 26, 110–117. doi: 10.1016/j.chb.2009.09.001

[ref36] Park O. C. Hopkins R. (1992). Instructional conditions for using dynamic visual displays: a review. Instr. Sci. 21, 427–449. doi: 10.1007/BF00118557

[ref37] Pi Z. Xu K. Liu C. Yang J. (2020). Instructor presence in video lectures: eye gaze matters, but not body orientation. Comput. Educ. 144:103713. doi: 10.1016/j.compedu.2019.103713

[ref38] Rayner K. Pollatsek A. Liversedge S. P. Reichle E. D. (2009). Eye movements and non-canonical reading: comments on. Vis. Res. 49, 2232–2236. doi: 10.1016/j.visres.2008.10.013, PMID: 19000705 PMC2746070

[ref39] Reinhold F. Hoch S. Werner B. Richter-Gebert J. Reiss K. (2020). Learning fractions with and without educational technology: what matters for high-achieving and low-achieving students? Learn. Instr. 65, –101264. doi: 10.1016/j.learninstruc.2019.101264

[ref40] Rey G. D. Beege M. Nebel S. Wirzberger M. Schmitt T. H. Schneider S. (2019). A meta-analysis of the segmenting effect. Educ. Psychol. Rev. 31, 389–419. doi: 10.1007/s10648-018-9456-4

[ref41] Richter J. Scheiter K. Eitel A. (2016). Signaling text-picture relations in multimedia learning: a comprehensive meta-analysis. Educ. Res. Rev. 17, 19–36. doi: 10.1016/j.edurev.2015.12.003

[ref42] Rieber L. P. (1990). Animation in computer-based instruction. Educ. Technol. Res. Dev. 38, 77–86. doi: 10.1007/BF02298250

[ref43] Schneider S. Beege M. Nebel S. Rey G. D. (2018). A meta-analysis of how signaling affects learning with media. Educ. Res. Rev. 23, 1–24. doi: 10.1016/j.edurev.2017.11.001

[ref44] Schotter E. R. Tran R. Rayner K. (2014). Don’t believe what you read (only once): comprehension is supported by regression during reading. Psychol. Sci. 25, 1218–1226. doi: 10.1177/0956797614531148, PMID: 24747167

[ref45] Seufert T. Brünken R. (2006). Cognitive load and the format of instructional aids for coherence formation. Appl. Cogn. Psychol. 20, 321–331. doi: 10.1002/acp.1248

[ref46] Smith B. Shimeld S. (2014). Using pictorial mnemonics in the learning of tax: a cognitive load perspective. Higher Educ. Res. Dev. 33, 565–579. doi: 10.1080/07294360.2013.841652

[ref47] Spanjers I. A. E. van Gog T. van Merriënboer J. J. G. (2010). A theoretical analysis of how segmentation of dynamic visualizations optimizes students’ learning. Educ. Psychol. Rev. 22, 411–423. doi: 10.1007/s10648-010-9135-6

[ref48] Stiller K. D. Petzold K. Zinnbauer P. (2011). Presentation time concerning system-paced multimedia instructions and the superiority of learner pacing. Australas. J. Educ. Technol. 27, 693–708. doi: 10.14742/ajet.945

[ref49] Treisman A. Schmidt H. (1982). Illusory conjunctions in the perception of objects. Cogn. Psychol. 14, 107–141. doi: 10.1016/0010-0285(82)90006-8 7053925

[ref50] Tso T. Y. Lu F. L. Tzeng S. C. Wu H. M. Chen M. J. Tan T. (2011). The effect of reducing task complexity on the reading of geometric proofs by experts and novices in a segmented way. N.C. J. Educ. Psychol. 43, 291–314. doi: 10.6251/BEP.20110517

[ref51] Tversky B. Heiser J. Mackenzie R. Lozano S. Morrison J. (2008). “Enriching animations,” in Learning with animation: Research implications for design. eds. Lowe R. K. Schnotz W. (Cambridge, UK: Cambridge University Press), 263–285.

[ref52] Van Gog T. (2014). The signaling (or cueing) principle in multimedia learning. Mayer R. E., Cambridge Handbook of Multimedia Learning, Cambridge, UK: Cambridge University Press, 263–278.

[ref53] Wang F. Duan Z. Zhou Z. (2013). Attention guidance in multimedia learning: the role of cueing. Adv. Psychol. Sci. 21, 1430–1440. doi: 10.3724/SP.J.1042.2013.01430

[ref54] Wolfe J. M. Horowitz T. S. (2004). What attributes guide the deployment of visual attention and how do they do it? Nat. Rev. Neurosci. 5, 495–501. doi: 10.1038/nrn1411, PMID: 15152199

[ref55] Wouters P. Paas F. van Merriënboer J. J. (2008). How to optimize learning from animated models: a review of guidelines based on cognitive load. Rev. Educ. Res. 78, 645–675. doi: 10.3102/0034654308320320

[ref56] Yantis S. Jonides J. (1996). Attentional capture by abrupt onsets: new perceptual objects or visual masking? J. Exp. Psychol. Hum. Percept. Perform. 22, 1505–1513. doi: 10.1037//0096-1523.22.6.1505 8953232

